# Spontaneous cerebrospinal fluid rhinorrhea: A case report and analysis: Erratum

**DOI:** 10.1097/MD.0000000000009954

**Published:** 2018-02-16

In the article, “Spontaneous cerebrospinal fluid rhinorrhea: A case report and analysis”^[1]^, which appears in Volume 97, Issue 5 of *Medicine*, the hospital name for the affiliations appears incorrectly and the affiliations should appear as:

^a^First Department of Neurosurgery, ^b^Department of Rheumatology, ^c^Department of Clinical Laboratory Diagnostics, China-Japan United Hospital of Jilin University, Changchun, Jilin Province, China.

The corresponding author's address should be Yu Fei Gao, First Department of Neurosurgery, China-Japan United Hospital of Jilin University, 126 Xiantaida Street, Changchun 130033, Jilin Province, China.

The study was approved by the ethics committee of China-Japan United Hospital of Jilin University.

The abstract should appear as:

**Rationale:** We report a rare case of Spontaneous cerebrospinal fluid rhinorrhea.

Patient concerns: The patient presented with a clear liquid nasal discharge twice.

**Diagnoses:** Nasal fluid test, head computed tomography and Magnetic resonance cisternography showed findings suggestive of cerebrospinal fluid rhinorrhea.

**Interventions:** Patient was treated with a transnasal transsphenoidal neuroendoscopic approach surgery for the repair of the cerebrospinal fluid leak.

Outcomes: No CSF rhinorrhea was observed after the surgery

**Lessons:** Spontaneous cerebrospinal fluid rhinorrhea is a rare disease, which is usually caused by the combination of a pre-existing weakening of the meninges and sudden violence. In patients with no history of trauma, nasal leakage is often overlooked. In our case, we diagnosed it accurately and successfully cured this patient with a transnasal transsphenoidal neuroendoscopic surgery approach.
