# Spontaneous cerebrospinal fluid rhinorrhea: A case report and analysis: Erratum

**DOI:** 10.1097/MD.0000000000010274

**Published:** 2018-03-23

In the article, “Spontaneous cerebrospinal fluid rhinorrhea: A case report and analysis”,^[1]^ which appears in Volume 97, Issue 5 of *Medicine*, the hospital name for the affiliations appears incorrectly in the previous erratum. United in the hospital name should be Union. The corrected information is:

^a^First Department of Neurosurgery, ^b^Department of Rheumatology, ^c^Department of Clinical Laboratory Diagnostics, China-Japan Union Hospital of Jilin University, Changchun, Jilin Province, China.

The corresponding author's address should be Yu Fei Gao, First Department of Neurosurgery, China-Japan Union Hospital of Jilin University, 126 Xiantaida Street, Changchun 130033, Jilin Province, China.

The study was approved by the ethics committee of China-Japan Union Hospital of Jilin University.

## References

[1] Chen G-Y Ma L Xu M-L Spontaneous cerebrospinal fluid rhinorrhea: A case report and analysis. *Medicine*. 2018 97:e9954. 29384861 10.1097/MD.0000000000009758 PMC5805433

